# Highly Efficient Oxygen Reduction N-Doped Carbon Nanosheets Were Prepared by Hydrothermal Carbonization

**DOI:** 10.3390/molecules29010003

**Published:** 2023-12-19

**Authors:** Yuchen Liu, Yajie Zheng, Peiyun Zhang, Junhua Hou

**Affiliations:** 1 School of Physics and Information Engineering, Shanxi Normal University, No.339 Taiyu Road, Xiaodian District, Taiyuan 030031, China; lh1304449229@163.com (Y.L.); 16635719311@163.com (Y.Z.); p1342433735@163.com (P.Z.); 2 Extreme Optical Collaborative Innovation Center, Shanxi University, No. 92, Wucheng Road, Xiaodian District, Taiyuan 030006, China; 3 Modern College of Humanities and Sciences, Shanxi Normal University, No.501 Binhe West Road, Yaodu District, Linfen 041000, China

**Keywords:** biomass, N-doped porous carbon, metal-free catalyst, ORR

## Abstract

A metal-free carbon catalyst is a kind of oxygen reduction catalyst with great prospects. It is an important material with potential to replace the traditional Pt catalyst. In this paper, a kind of irregular and ultra-thin carbon nanosheet (K180M-300-900) with high catalytic activity was synthesized by hydrothermal calcination using okra as a biomass and NH_4_Cl as an N source. The prepared nitrogen-doped metal-free catalyst with high pyridine-N and graphitic-N provides an extremely large number of active sites and has certain lattice defects. Ultra-thin carbon nanosheets promote sufficient contact between the catalyst and electrolyte, promote the diffusion of oxygen, and result in a faster transfer rate of electrons. The initial potential and half-slope potential of K180M-300-900 are 0.99 V and 0.82 V, respectively, which are comparable to those of 20% Pt/C. In addition, the stability and methanol tolerance of this catalyst (K180M-300-900) are better than 20% Pt/C, so it has great development potential and application value. This result provides a new method to prepare metal-free carbon materials that will take the place of traditional Pt catalysts.

## 1. Introduction

Serious environmental pollution and rapid energy consumption are the great challenges faced by mankind at present, so the development of and search for new energy is an important subject of human research [1,2,3]. There is an urgent need to find clean and renewable energy technologies. For example, the fuel cell is a new type of energy supply device that is able to convert chemical energy directly into electricity and is not restricted by the Carnot cycle [4,5,6]. The fuel cell has a high utilization rate and low pollution emission, and has great application potential, so it has been widely researched by mankind [7]. The core of new energy technologies for fuel cells is ORR, and it is the key to realizing its commercial application, indicating that the development of high-performance catalytic materials has become a new technical challenge [8,9]. At present, among ORR electrochemical catalysts, Pt has good properties such as molecular adsorption, dissociation, and so on, making it universally recognized as the catalyst with the best performance, and it is commonly used as a commercial catalyst in batteries. However, its low storage capability and high price in China, are large obstacles to the commercialization of Pt on a large scale [10,11,12]. Therefore, finding green and efficient alternatives is one of the main tasks of fuel cell technology.

Up to now, people have developed a large number of metal oxides, nitrides, and non-precious metal catalysts to replace the Pt catalyst. Among them, doped carbon materials such as heteroatoms (N, P, S) may produce unexpected results in terms of ORR performance [13,14]. In the periodic table, C and N are adjacent to each other and have similar atomic radii, making N one of the most promising candidates for doping [15]. The spin density and charge distribution of sP^2^ carbon are changed by the nitrogen-doped carbon material, resulting in more defect sites and active sites, which increases the catalytic activity [16,17,18,19,20]. In conclusion, nitrogen-doped carbon catalysts have far more excellent properties, so it is helpful as a way to improve the efficiency and stability of ORR. Recently, Xu et al. synthesized an N-doped carbon catalyst with a specific surface area (SSA) up to 1010 m^2^/g by using white grass as the raw material through hydrothermal and nitrogen doping [21]. Jiang et al. adopted a spontaneous gas foaming strategy and synthesized ultra-thin carbon nanosheets (NCNs) with a specific surface area (SSA) up to 1793 m^2^/g N by carbonizing citric acid and NH_4_Cl in a simple step [22]. Li et al. used Acorus as the precursor and ammonium chloride as the N source. After calcination, a hydrothermal reaction kettle was used to heat the final product, which had excellent ORR activity [23]. Although great progress has been made, carbon-based catalysts remain a major challenge due to their complex processes and catalytic properties [24]. Therefore, it is very necessary to select suitable ideal biomass and simplify the process to design porous carbon nanomaterial catalysts with good ORR activity for nitrogen doping [25,26,27,28].

Based on the above reasons, we proposed a simple method, using okra as the C source and NH_4_Cl as the N source, through the hydrothermal calcination method to synthesize a high-performance ORR biomass carbon material catalyst. Okra was used as the C source because of its special internal structure, and the mucus is full of polysaccharides, so the mature okra is a large molecule of many glucose linked together. The final product K180M-300-900 carbon skeleton is rich in graphitic nitrogen and pyridine nitrogen, thus providing an extremely large number of active sites, making the initial potential and semi-slope potential of 0.99 V and 0.82 V, respectively, comparable to those of 20% Pt/C. K180M-300-900 shows excellent catalytic activity, methanol resistance, and stability in alkaline electrolyte solutions, which are considered important factors for ORR [29,30].

The oxygen reduction reaction (ORR) is an important cathode reaction in a series of energy applications. The ORR reaction path in an aqueous solution is divided into two categories. One is the 4e^−^ reduction path of direct reaction; the other is the 2e^−^ reduction path with intermediate products, and the specific reaction path depends on the type of catalyst, and the reaction mechanism in different acid-base solutions is different [31,32].

The reaction mechanism in an alkaline environment is as follows:

Four−electron path:

$$O_{2}+2H_{2}O+4e^{-}\to 4OH^{-}$$

Two−electron path:

$$O_{2}+H_{2}O+2e^{-}\to HO_{2}^{-}+OH^{-}$$

$$HO_{2}^{-}+H_{2}O+2e^{-}\to 3OH^{-}$$

The reaction mechanism in an acidic environment is as follows:

Four−electron path:

$$O_{2}+4H^{+}+4e^{-}\to 2H_{2}O$$

Two−electron path:

$$H_{2}O_{2}+2H^{+}+2e^{-}\to 2H_{2}O$$

## 2. Results and Discussion

### 2.1. Surface Morphology Characterization of Catalyst

**Figure 1:**
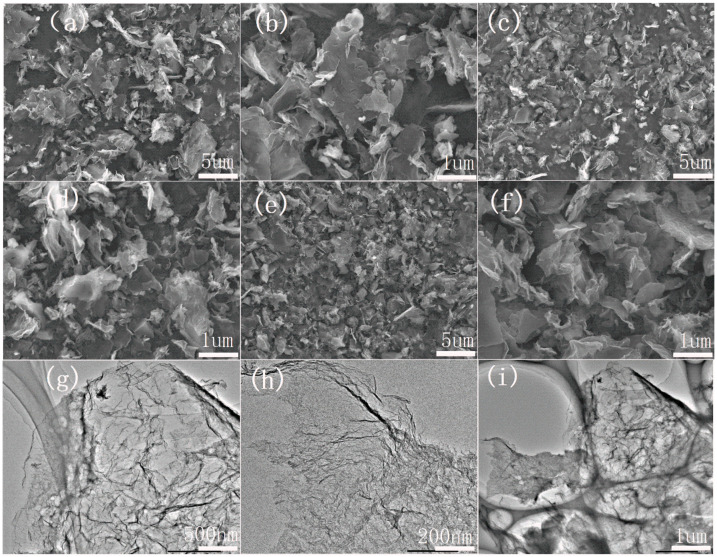
SEM images of K-300-900 (**a**,**b**), K180M-900 (**c**,**d**), and K180M-300-900 (**e**,**f**). TEM image of K180M-300-900 (**g**–**i**).

The scanning electron microscope (SEM) and transmission electron microscope (TEM) morphologies of electrocatalysts made from the same substance by different processes are shown in Figure 1. According to SEM, undoped carbon materials K-300-900 (Figure 1a,b) have smooth and flat carbon nanosheets, indicating that this special morphology is related to the internal structure of okra itself. K180M-900 (Figure 1c,d) has thinner carbon nanosheets with a small amount of pore structure, but K180M-300-900 (Figure 1e,f) has a significantly irregular pore structure and thinner carbon nanosheets. Compared with K-300-900 and K180M-900, K180M-300-900 is sparse, fluffy, thinner, and more abundant in pores, corresponding to the result of the specific surface area, which is attributed to the increased acidity of NH_4_Cl decomposition with the increase in temperature in the hydrothermal process, thus destroying the internal structure. With the addition of the N element, the biomass surface is opened and many pore structures are left in the body. In the carbonization process, the pre-carbonization at 300 °C makes the pore structure maintain stability. According to the TEM image of K180M-300-900 (Figure 1g,h), the flaky folds also confirm that the internal structure is dominated by sparse porous and irregular carbon nanosheets, the morphology is disordered, and the surface structure is amorphous. It is further demonstrated in Figure 1, in the high-resolution image K180M-300-900, (Figure 1i) that the surface of the internal structure is “open” with a large number of holes in the pore structure. Therefore, the addition of the N element and pre-carbonization at a low temperature can effectively promote the formation of carbon nanosheets on the catalyst surface and the appearance of internal microporous structures, thus increasing the accessibility of active sites and the diffusion rate of ions in the electrochemical reaction of the catalyst.

### 2.2. Structural Characterization of Catalysts

**Figure 2:**
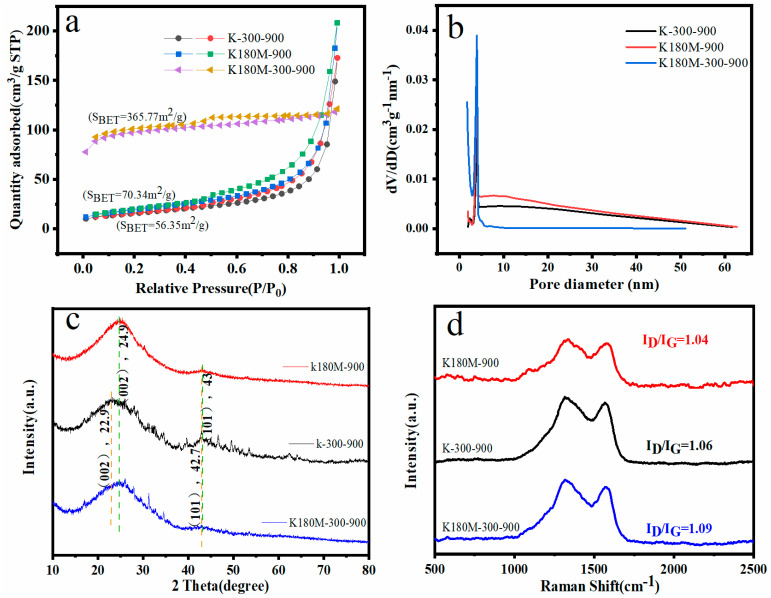
N_2_ adsorption–desorption curve (**a**), pore size distribution curve (**b**), XRD pattern (**c**), and Raman spectrum (**d**) of the specific surface areas of K180M−300−900, K180M−900, and K300−900.

In order to further study the internal structure of the catalyst, a nitrogen adsorption desorption curve at 77 K was tested and the pore size distribution was analyzed. As shown in Figure 2, in the relative pressure range of 0.45–1.0 P/P_0_, the adsorption desorption curves of K-300-900 and K180M-900 show a type IV isotherm with an obvious H3 type hysteresis curve, which is due to the presence of mesoporous structures in the material and no adsorption saturation in the higher relative pressure region. K180M-300-900 has an obvious H4 hysteresis loop in the high-pressure region (P/P_0_ > 0.4), which is a composite of type I and type II adsorption isotherms, reflecting the presence of a mesoporous structure. The rapid absorption of N_2_ by K180M-300-900 at a low pressure (P/P_0_ < 0.45) indicates the role of micropores. As can be seen from Figure 2a, the specific surface area of K180M-300-900 (365.77 m^2^/g) is much larger than those of K180M-900 (70.34 m^2^/g) and K-300-900 (56.35 m^2^/g), and the increase in S_BET_ is related to the establishment of porosity. It can be further analyzed from Figure 2b that the pore size distribution curve of Barrett–Joyner–Halenda (BJH) confirms that the catalyst is mainly mesoporous. It can be seen from Figure 2b that the pore size distribution centers of K180M-300-900, K180M-900, and K-300-900 are mainly 3.9 nm mesoporous, and their pore distribution ranges from 2 nm to 20 nm. As we all know, having a large specific surface area and mesoporous structure will help the target molecule to enter the inner surface and make a fuller contact with the catalyst, thereby improving the catalytic activity and further building the electron transport and transfer channel. The porous structure of K180M-300-900 is conducive to the electron transport and transfer during the electrochemical reaction process, and is conducive to the improvement in its ORR.

All catalysts were X-ray diffracted using X-ray diffraction (XRD), and their crystal structures were analyzed. As shown in Figure 2c, K180M-300-900 and K180M-900 appear as two wide diffraction peaks near the center of 24.9 °C and 43 °C, representing the (002) and (101) planes (33) of graphitic carbon in biological carbon, respectively. Table 1 uses the XRD pattern to calculate that the Crystallite Size Ds (nm) of K180M-300-900, K180M-900, and K-300-900 are 0.52 nm, 0.57 nm, and 0.73 nm, respectively, at the (002) plane. It is further explained that K180M-300-900 has a wide diffraction peak in the (002) plane, which is a typical feature of carbon materials with a low degree of graphitization. At the same time, the carbon peaks corresponding to the (002) and (101) planes of K-300-900 are near 22.9 °C and 42.7 °C, and the crystal surface of (002) has a significant shift to the left. The reason for this phenomenon is that K180M-300-900 and K180M-900 have the addition of heteroatom N. As a result, the lattice spacing becomes larger and defects increase. XRD results show that the presence of graphitic carbon is confirmed, which makes the sample have good electrical conductivity. Heteroatom doping can lead to the disordered carbon structure of the catalyst, which is formed by heteroatom inhibiting the carbon structure sp^2^-C.

A Raman spectrometer can obtain molecular structure information based on the Raman effect. In the Raman diagram, there are two characteristic peaks of C-atom crystals, namely, peak D and peak G. Peak D reflects the C defect of the lattice, while peak G reflects the degree of carbonization of the material [33]. As is shown in Figure 2d, peak D and peak G of all catalysts are around 1315 cm^−1^ and 1373 cm^−1^, respectively. The I_D_/I_G_ is the intensity ratio between peak D and peak G, and the larger the ratio is, the more defects there are of the C atomic crystal [34,35]. The I_D_/I_G_ ratios of K180M-300-900, K-300-900, and K180M-900 are 1.09, 1.06, and 1.04, respectively, and the value of K180M-300-900 is the largest, indicating that it has the most crystal defects. The reason for this is that after hydrothermal treatment and high-temperature calcination, the N element in NH_4_Cl is fully doped into the active site, resulting in larger crystal defects. This measurement result is consistent with the previous XRD measurement results. It is confirmed that the introduction of the N element plays an important role in crystal defects’ specifically, their surface area and pore size.

### 2.3. Characterization of Surface Functional Groups of Catalysts

**Figure 3:**
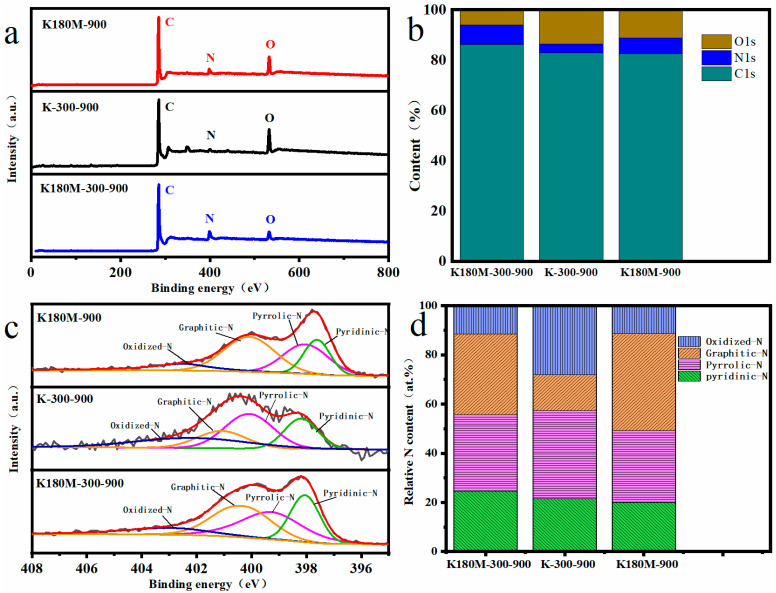
XPS spectra of K180M-300-900, K180M-900, and K-300-900 (**a**), relative contents of C, N, and O (**b**), high-resolution XPS spectra of N1s (**c**), and relative contents of N species (**d**).

X-ray photoelectron spectroscopy (XPS) was used to analyze the elements of all the catalysts, and the results show that C1s, N1s, and O1s coexist, with peak centers around 285, 399, and 532 eV, respectively, as shown in Figure 3a [36]. According to the elemental analysis, the elemental contents of the C, N, O, and N species (pyridine-N, pyrrole-N, graphite-N, oxide-N) of each catalyst are listed in Table 2. Compared with K-300-900 (3.38%), the N contents of K180M-300-900 (7.74%) and K180M-900 (6.23%) are significantly increased, which proves that NH_4_Cl, as the N donor of the catalyst, effectively increased the N doping active site in the hydrothermal process stage. Figure 3b shows the contents of C, N, and O in all catalysts. According to the column chart, it can be intuitively shown that the contents of C and N in K180M-300-900 are more than those in K180M-900, indicating that the intermediate insulation temperature of 300 °C is one of the important factors affecting the catalyst structure in the carbonization process. Figure 3c shows the high-resolution N1s spectra of all catalysts. K180M-300-900, K-300-900, and K180M-900 show four characteristic peaks due to the doping of the N element in the range of 398.10–398.84, 399.54–399.89, 400.33–400.82, and 401.39–402.15 eV, corresponding to pyridine-N, pyrrole-N, graphite-N, and oxide-N, respectively [37].

Some studies have shown that pyridine-N and graphite-N have greater effects on the ORR activity of catalysts without metals [38,39]. This conclusion is consistent with the increase in the atomic content of the N element in Table 2 from K-300-900 (3.38%) to K180M-900 (6.23%) and K180M-300-900 (7.74%). According to Table 2 and Figure 3d, compared with K180M-300-900, the pyridine-N content of K180M-900 increased from 20% to 24.54%, while the graphite-N content decreased from 39.37% to 32.74%. The increase in pyridine-N content and the decrease in graphite-N content confirmed that the reaction was mainly a four-electron process. This is consistent with the results of the previous Raman test. Figure S1 shows that 284.7, 285.8, and 289 eV are the central characteristic peaks at three positions of the high-resolution C1s spectrum, corresponding to C=C (sp^2^), C-N, and C-O, respectively. Similarly, Figure S2 shows two characteristic peaks centered at 531.5 and 533.5 eV in high-resolution O1s spectra, corresponding to O=C and O-C, respectively. These results all indicate that species N is embedded in the skeleton of K180M-300-900, resulting in a large number of active sites and structural defects.

### 2.4. Electrochemical Properties of Catalysts

**Figure 4:**
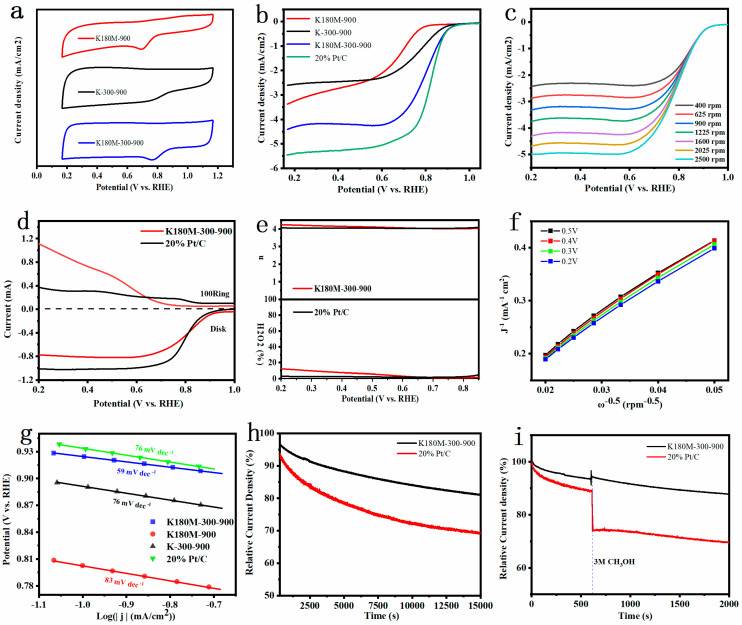
CV curves were obtained when all catalysts were in 0.1 M KOH solution, and were saturated with O₂ in the condition of indoor temperature and scan rate of 50 mV/s (**a**). LSV of all catalysts were measured at an electrode rotation rate of 1600 rpm and a scan rate of 10 mV/s (**b**). LSV curves of K180M−300−900 were obtained at different rotation rates from 400 to 2500 rpm (**c**). The measured RRDE linear scanning voltammogram (**d**) was obtained at an electrode rotation rate of 1600 rpm and a scan rate of 5 mV/s. Electron transfer number n and H_2_O_2_ yield were calculated from K180M−300−900 and 20% Pt/C RRDE measurements (**e**). K180M−300−900 K—L diagram (**f**) was calculated according to different speeds and Tafel plot of all catalysts (**g**). Stability curves for K180M−300−900 and 20% Pt/C in O_2_-saturated 0.1 M KOH solution (**h**) and durability test for methanol (**i**) were obtained.

In order to evaluate the performance of all catalysts, we used the Shanghai Chenhua CHI760E electrochemical workstation and Metrohm Rotating Electrode to test their performance. First, cyclic voltammetry and linear sweep voltammetry tests were performed on all catalysts at room temperature and in a solution of 0.1 M KOH saturated with O_2_. According to Figure 4a, in the electrolyte solution saturated with O_2_, the CV curves of K180M-300-900 and K180M-900 have obvious oxygen reduction peaks, and the reduction peak of K180M-300-900 is corrected, but the reduction peak of K-300-900 does not appear. By comparing the peak potential of RHE, it can be clearly seen that the activity of the three catalysts follows the sequence that K180M-300-900 is greater than K180M-900, and K180M-900 is greater than K-300-900. According to Figure 4b, the initial potential of K180M-300-900 is 0.99VvsRHE, which is greatly improved compared with K-300-900 (0.95VvsRHE) and K180M-900 (0.96VvsRHE). The order of half-slope potential is: K180M-300-900 (0.82 V) is greater than K-300-900 (0.74 V), and K-300-900 (0.74 V) is greater than K180M-900 (0.67 V), and compared with the other two catalysts, K180M-300-900 has a larger limiting current density that is close to the performance of commercial 20% Pt/C catalysts. The limiting current density is heavily dependent on mass transport within the reaction zone and is closely related to the properties of mesoporous pores, a result corresponding to Figure 2a,b. K180M-300-900 has better performance due to its large specific surface area, more structural defects caused by nitrogen doping, and increased content of pyridine-N and graphite-N. As a reference, the ORR performance test results of other recent research groups on metal-free carbon-based catalysts are summarized, as shown in Table 3. It is worth noting that the activity of the K180M-300-900 ORR catalyst is comparable to the reported metal-free C material catalysts and commercial 20% Pt/C catalysts.

Figure 4c shows the LSV curve of K180M-300-900 at different speeds ranging from 400 to 2500 rpm. The results show that the initial potential remains unchanged, and the limiting current density increases with the increase in the speed. This is because the faster the electrode speed is, the thinner the diffusion layer on the electrode surface is, and the reactant in the solution can diffuse faster to the electrode surface, resulting in a greater limiting current. In addition, according to the Koutechy–Levich (K–L) equation, we processed the data in Figure 4c to obtain Figure 4f. The results show that K180M-300-900 is linear and parallel at different potentials, confirming that it is a first-order reaction kinetics in an ORR reaction [34]. The electron number n of K180M-300-900 is in the range of 3.88~4.0, which proves that K180M-300-900 mainly follows the four-electron reaction [46]. As shown in Figure 4d, both K180M-300-900 and 20% Pt/C show a slight ring current density, indicating that there is only a small amount of H_2_O_2_ at the ring electrode, further confirming that K180M-300-900 has a higher catalytic performance. The electron transfer number n and H_2_O_2_ in Figure 4e are calculated. The results show that K180M-300-900 is a quasi-four-electron reaction, which is consistent with the conclusion obtained from the K-L equation. The Tafel slope is an important index reflecting reaction dynamics. That is, reactions with different Tafel slopes have different speed control steps. In Figure 4g, compared with K-300-900 (76 mVdec^−1^), K180M-900 (83 mVdec^−1^), and 20% Pt/C (76 mVdec^−1^), the minimum value of K180M-300-900 is 59 mVdec^−1^, which means that the current density increases faster. The smaller the electron transfer barrier is, the faster the transfer speed is, and the corresponding performance is better, which further indicates that K180M-300-900 has excellent electrocatalytic activity [47]. At the same time, as shown in Figure S2, the results of electrochemical impedance spectroscopy (EIS) show that the charge-transfer resistance (Rct) of K180M-900, K-300-900, and K180M-300-900 decreases successively to 871.1, 828.9, and 234.7 Ω. This indicates in the ORR reaction of K180M-300-900 that the transfer speed of charge is faster [48].

It is well known that designing and preparing metal-free carbon catalysts with high durability and high methanol resistance is a difficult task. As shown in Figure 4h, K180M-300-900 and 20% Pt/C were tested through the current time curve. After 15,000 s, K180M-300-900 maintains 81.1% of the initial current, and Pt/C maintains 72.5% of the initial current. The results show that the stability of K180M-300-900 is better than 20% Pt/C, which can meet the practical requirements. In order to test the methanol resistance of the catalyst, 5 ml of anhydrous methanol was injected into the solution at 600 s. It was clearly observed that in the 2000 s, the initial currents of K180M-300-900 and 20% Pt/C remained at 87.8% and 70.1%, respectively. Figure 4i shows that K180M-300-900 has better effects of avoiding methanol cross-poisoning. K180M-300-900 is a metal-free carbon catalyst with high durability and methanol resistance, which has potential and is promising.

From the above discussion, it can be concluded that the reasons why K180M-300-900 has excellent ORR performance are that: (1) it has sparse, fluffy, wrinkled carbon nanosheets, and a large specific surface area that promote electrolyte contact and accelerate O_2_ diffusion [22]; (2) the doping of the N atom, the content of pyridine -N, and graphite -N increases, thus speeding up the charge transfer rate; and (3) a large number of exposed edges and crystal defects act as active sites, which significantly improve the catalytic performance [49].

## 3. Experimental Part

### 3.1. Chemicals and Materials

Okra as a precursor is harvested from Shenzhen, Guangdong Province, China. NH_4_Cl, Pt/C (20 wt% platinum carbon catalyst) is purchased from Shanghai Maclean Biochemical Co. Ethanol is purchased from Tianjin Beichen Founder Reagent Factory. Nafion solution (5 wt%) is from Shanghai Sanmus Industrial Co. All chemical reagents are of analytical grade and used directly without further purification.

### 3.2. Synthesis of Materials

The okra was cut into small pieces, rinsed repeatedly with deionised water, washed and placed in a constant temperature oven at 70 °C for 48 h, to obtain dehydrated okra, which was then uniformly crushed with a pulveriser at a speed of 32,000 r/min for 10 min, to obtain a uniform 100 mesh okra powder named K precursor (K stands for okra, the same below). The mass ratio of K precursor to ammonium chloride (1:5) was taken, and 50 mL of deionized water was added for ultrasound until uniform distribution (M represents NH_4_Cl). Transfer it to a stainless steel high pressure reactor (100 mL) lined with tetrafluoroethylene and heat it at 180 °C for 15 h using an oven, named K180M. Then, transfer the sample to the constant temperature oven, 70 °C drying. The dried samples were fully ground in an agate mortar and placed in a tubular furnace under N_2_ atmosphere at a heating rate of 5 °C/min to 300 °C for 2 h, followed by 900 °C for 2 h of carbonization to obtain K180M-300-900 N-doped carbon material.

Similarly, the sample K180M after hydrothermal drying is heated to 900 degrees for 2 h to carbonize it, and K180M-900 N-doped carbon material is obtained. The okra without hydrothermal treatment is directly heated to 300 degrees and 900 degrees for two hours respectively without adding NH_4_Cl, and K-300-900 carbon material is obtained. The heating rate is 5 °C/min. Finally, a series of carbonized materials were fully ground with an agate mortar, washed repeatedly with deionized water and anhydrous ethanol to remove soluble impurities, and dried at 60 °C.

### 3.3. Structural Characterization

The catalyst was characterized using a field emission scanning electron microscope (SEM, model: Regulus 8100, made in Japan, Brand: Hitachi, manufacturer: Hitachi Scientific Instruments (Beijing) Co., Ltd., Beijing, China) and a transmission electron microscope (TEM, Model: JEM-2800, Made in Japan, Brand: Japan Electronics, Manufacturer: JEOL Japan Electronics Co., Ltd., Tokyo, Japan). The degree of graphitization and crystal structure of the catalysts were probed using X-ray diffractometry (XRD, model MPD, Made in Europe, Brand: Malvern Panaco, manufacturer: Panaco, Netherlands) and Raman spectroscopy (model: LabRam HR Evolution, made in France, brand: HORIBA JY, manufacturer: Shanghai Juna Technology Co., LTD). The elemental composition and content of the catalyst were investigated using X-ray photoelectron spectroscopy (model: Thermo Kalpha, made in the UK, brand: Thermo Fisher, manufacturer: Thermo Fisher Technologies Ltd., Waltham, MA, USA). The specific surface area and the porosity and pore size distribution of the catalyst were measured by a fully automated specific surface and porosity analyser BET (model: Mac ASAP 2460, made in USA, Brand: Mack Instruments, manufacturer: McMuritik (Shanghai) Instruments Co., Ltd., Shanghai, China).

### 3.4. Electrochemical Tests

Firstly, we polished the working electrode with 0.03 mm aluminium oxide on buckskin until it was smooth and shiny, and then tested it in a potassium ferricyanide and potassium chloride solution. When the voltage subtraction value of the oxidation and reduction peaks across the measurement cycle voltammetry (CV) was around 100, it proved that the working electrode had been polished well. Then it was cleaned by DI water, gently wiped with alcohol, and dried naturally and was ready for use. Then 5 mg of the catalyst powder was ground in an agate mortar, and 50 μL of Nafion solution, 250 μL of isopropanol solution, and 700 μL of deionized water were taken and placed in a 1.5 mL centrifuge tube and sonicated for 1 h using an ultrasonic instrument to make a catalyst suspension. Finally, a pipette gun was used to take 10 μL of the catalyst solution to the centre of the working electrode and to dry it naturally (the average catalyst loading was 0.25 mg·cm^−2^). All electrochemical tests were carried out at a room temperature of 25 °C in a 0.1 M KOH electrolyte. In order to saturate the solution with oxygen, oxygen was passed for half an hour before each measurement was carried out. Cyclic voltammetric curves (CV) were measured in an oxygen-saturated solution at a scan rate of 50 mV/s over a potential range of −0.8 to 0.2 V, and, similarly, linear scanning voltammetric curves (LSV) were measured in this solution at a scan rate of 10 mV/s over a potential range of −0.8 to 0.2 V from 400 rpm to 2500 rpm. All potentials were converted to the standard reversible hydrogen electrode (RHE) potential with the following equation:

$$E_{\left(\mathrm{RHE}\right)}=E_{\left(\mathrm{Ag}/\mathrm{AgCl}\right)}+E_{\left(\mathrm{Ag}/\mathrm{AgCl}\right)}^{\theta }+0.0591\times \mathrm{pH}$$

The slope and intercept of the Koutecky-Lecich (K-L) curve at potentials of 0.2 V–0.6 V (vs RHE) were calculated by linear fitting to give the number of electron transfers n and the current density with the following equation [50]:

$$\frac{1}{J}=\frac{1}{J_{K}}+\frac{1}{J_{L}}=\frac{1}{J_{K}}+\frac{1}{B\omega^{1/2}}$$

$$B=0.62nFC_{0}D_{0}^{2/3}v^{-1/6}$$

$$J_{k}=nFkC_{0}$$

J represents the actual current density; J_K_ represents the kinetic limit diffusion current density; w is the working electrode rotation speed; n is the number of electron transfers in ORR measurements; and F is the Faraday constant; C_0_ (C_0_ = 1.2 × 10^−6^ mol·cm^−3^) and D_0_ (D_0_ = 1.9 × 10^−5^ cm^2^·s^−1^) are the oxygen concentration and diffusion coefficient of oxygen in 0.1 M KOH solution, respectively; V is the kinematic viscosity of the electrolyte (1.0 × 10^−2^ cm^2^·s^−1^); k is the electron-transfer rate constant. The number of electron transfers from the catalyst to the ORR and the yield of hydrogen peroxide in an O_2_-saturated 0.1 M KOH solution at 1600 rpm and a scan rate of 50 mV/s were measured using a rotating ring-disk electrode (RRDE). The equations for the number of electrons transferred (n) and the yield of hydrogen peroxide H_2_O_2_ are as follows [33]:

$$n=\frac{4I_{D}}{I_{D}+(I_{R}/N)}$$

$$\%HO_{2}^{-}=200\times \frac{I_{R}/N}{I_{D}+I_{R}/N}$$

I_D_ and I_R_ are plate and ring currents respectively. N = 0.37 is the collection efficiency of the Pt ring.

## 4. Conclusions

In this paper, using okra as precursor and NH_4_Cl as the N source, irregular and ultra-thin carbon nanosheets (K180M-300-900) with high pyridine-N and graphite-N were synthesized by the hydrothermal and calcined method. The smooth carbon nanosheets of K-300-900 are derived from the material structure of the biomass itself, while there are a large number of sparse, fluffy, porous, and thinner carbon nanosheets in K180M-300-900. The formation of this morphology may be due to the etching effect of gas produced in NH_4_Cl on the carbon matrix. This increases the number of active sites (see (1) and (2) for the chemical equation [51]), and at the same time, provides the N element for the carbon matrix under high-temperature calcination, forming a multi-pore structure on the outer surface and generating more N-related electrocatalytic active sites:

(1) $$NH_{4}Cl≜NH_{3}↑+HCl↑\;$$

(2) $$C(s)+NH_{3}(g)\to HCN(g)+H_{2}(g)\;$$

Through the testing of its morphology, crystal structure, element content, and properties, the results show that K180M-300-900 presents ultra-thin carbon nanosheets with a large specific surface area and high crystal defects, which is due to the increased acidity of the NH_4_Cl solution with the increase in temperature in the hydrothermal process, which corrodes and destroys the internal structure of the biomass. At the same time, the increase in the N element content further promotes the formation of a rich pore structure of carbon materials under the intermediate insulation temperature and high-temperature calcination, resulting in the formation of large lattice defects, thus increasing the specific surface area of the material. The LSV measurement shows that K180M-300-900’s initial potential and half-slope potential are 0.99 V and 0.82 V, respectively. In addition, the Tafel slope value of K180M-300-900 is less than that of 20% Pt/C, indicating that the electron transfer obstruction is small and the transfer speed is faster, and the stability and methanol resistance of K180M-300-900 is better than 20% Pt/C. This work provides a new method and strategy for developing a low-cost, pollution-free, and efficient metal-free carbon catalyst in an alkaline solution.

## Figures and Tables

**Table 1:** Raman and XRD results of the prepared activated carbons.

Samples	2θ Degree (002)	2θ Degree (101)	Crystalline Size (nm)	I_D_/I_G_ Ratio
K180M-300-900	24.8	43	0.52	1.09
K180M-900	24.6	43.1	0.57	1.06
K-300-900	22.9	42.7	0.73	1.04

**Table 2:** This shows the content of C, N, and O elements of the catalyst through elemental analysis and XPS calculation.

Samples	Elemental Content (at.%)			N Configuration (%)			
	C1s	N1s	O1s	Pyridinic-N	Pyrrolic-N	Graphitic-N	Oxidized-N
K180M-300-900	86.14	7.74	6.12	24.54	31.2	32.74	11.52
K-300-900	82.96	3.38	13.66	21.62	35.81	14.49	28.08
K180M-900	82.54	6.23	11.23	20	29.22	39.37	11.41

**Table 3:** ORR activity of different catalysts.

Catalyst	Onset Potential (V vs. RHE)	Half Wave Potential (V vs. RHE)	Reference
PP350KOH800-S	0.98	0.87	[40]
N/C-Np+NG	0.98	0.86	[41]
NBCNT-10	0.958	0.82	[42]
HHPT-900	0.83	0.75	[43]
T-NFC	0.81	0.66	[44]
N-PC	0.96	0.84	[45]
NCN-1000-5	0.95	0.82	[34]

## Data Availability

Data are contained within the article and supplementary materials.

## Supplementary Materials

The following supporting information can be downloaded at: https://www.mdpi.com/article/10.3390/molecules29010003/s1, Figure S1: High-resolution XPS of the C1s (a) and O1s (b) peaks of K180M-300-900, K180M-900, and K-300-900; Figure S2: Electrochemical impedance spectra (EIS) of K180M-300-900, K180M-900, and K-300-900 in saturated 0.1 M KOH solutions.
