# Supplementary material for: Highly Efficient Oxygen Reduction N-Doped Carbon Nanosheets Were Prepared by Hydrothermal Carbonization
Source: Molecules. 2023 Dec 19;29(1):3. doi: 10.3390/molecules29010003 (PMC10780226; doi:10.3390/molecules29010003)
Supplement: Supplementary file 1 [file molecules-29-00003-s001.zip › molecules-2758557- Supporting information.pdf]

Supporting information

Highly Efficient Oxygen Reduction N-Doped Carbon Nanosheets Were Prepared by Hydrothermal Carbonization

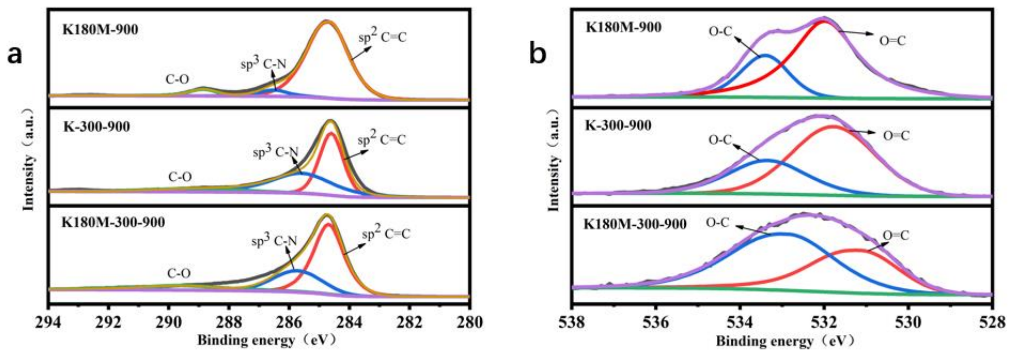

Figure S1. High-resolution XPS of the C1s (a) and O1s (b) peaks of K180M-300-900, K180M-900 and K-300-900

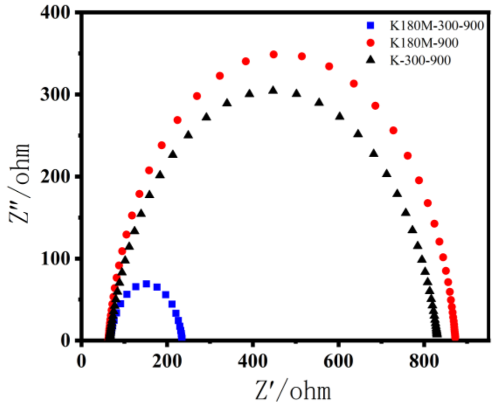

Figure S2. Electrochemical impedance spectra (EIS) of K180M-300-900, K180M-900 and K-300-900 in saturated 0.1 M KOH solutions
